# Syringaldehyde Alleviates Cardiac Hypertrophy Induced by Hyperglycemia in H9c2 Cells Through GLP-1 Receptor Signals

**DOI:** 10.3390/ph18010110

**Published:** 2025-01-16

**Authors:** Yingxiao Li, Chao-Tien Hsu, Ting-Ting Yang, Kai-Chun Cheng

**Affiliations:** 1 Department of Anatomy, College of Medicine, I-Shou University, Kaohsiung 824005, Taiwan; yxli0809@isu.edu.tw; 2 Department of Pathology, E-Da Hospital, I-Shou University, Kaohsiung 824005, Taiwan; ed103797@edah.org.tw; 3 School of Chinese Medicine for Post Baccalaureate, College of Medicine, I-Shou University, Kaohsiung 824005, Taiwan; tingting@isu.edu.tw; 4 Department of Pharmacy, College of Pharmacy and Health Care, Tajen University, Pingtung 90741, Taiwan

**Keywords:** AMPK, cardiac hypertrophy, diabetes complications, GLP-1 resistance, O-linked N-acetylglucosamine transferase (OGT), syringaldehyde

## Abstract

**Background:** Cardiac hypertrophy is a significant complication of diabetes, often triggered by hyperglycemia. Glucagon-like peptide-1 (GLP-1) receptor agonists alleviate cardiac hypertrophy, but their efficacy diminishes under GLP-1 resistance. Syringaldehyde (SA), a natural phenolic compound, may activate GLP-1 receptors and mitigate hypertrophy. This study explores SA’s therapeutic potential in hyperglycemia-induced cardiac hypertrophy in H9c2 cardiomyocytes. **Methods:** H9c2 cells were exposed to high glucose to induce hypertrophy. Cells were treated with varying SA concentrations, and hypertrophic biomarkers were analyzed using ELISA, qPCR, and Western blot. **Results:** SA reduced cell size and hypertrophic biomarkers in a dose-dependent manner while increasing GLP-1 receptor expression and cAMP levels. These effects were attenuated in GLP-1-resistant cells, highlighting the role of GLP-1 receptor activation. AMPK activation was essential, as its inhibition abolished SA’s effects. SA also decreased O-linked N-acetylglucosamine transferase (OGT) expression via AMPK activation, contributing to reduced hypertrophy. **Conclusions:** SA alleviates hyperglycemia-induced cardiac hypertrophy in H9c2 cells by activating the GLP-1 receptor and AMPK signaling pathway.

## 1. Introduction

Diabetic cardiomyopathy (DCM) is a common consequence of diabetes characterized by hypertrophy in cardiomyocytes in response to prolonged exposure to elevated glucose levels [1]. Cardiac hypertrophy is characterized by cellular hypertrophy resulting from age and/or neurohumoral stimulation, and it is regarded as an independent risk factor for heart failure. Consequently, it may be categorized into pathological or physiological processes [2,3,4]. Furthermore, atrial natriuretic peptide (ANP), brain/B-type natriuretic peptide (BNP), and β-myosin heavy chain (β-MHC) serve as markers of hypertrophy progression [5]. The goal of treatment for hypertrophic cardiomyopathy is to reduce the risk of heart failure. However, there has been no confirmed effective treatment for DCM [6].

Glucagon-like peptide-1 (GLP-1) is a member of the incretin hormone family, which is released from the stomach to induce insulin secretion after consuming nutrients [7]. Moreover, cardiomyocytes express the GLP-1 receptor (GLP-1R) [8]. GLP-1R activation has been extensively studied for its dual role in managing diabetes-related complications and reducing cardiac hypertrophy [9]. In clinical trials, GLP-1R agonists have been shown to stabilize and decrease blood glucose levels while also promoting weight loss [10]. Moreover, these agonists exhibit direct cardioprotective [11] and renal protective effects [12], achieved by reducing oxidative stress, improving myocardial energy efficiency, and modulating inflammatory pathways [13]. Recent studies have highlighted additional benefits of GLP-1R agonists. Semaglutide has been found to ameliorate cardiac remodeling in male mice by optimizing energy substrate utilization through the Creb5/NR4a1 axis [14]. Similarly, exendin-4 (EX-4) has been shown to reduce infarct size and myocardial stiffness in animal models of acute myocardial infarction [15]. Another GLP-1 agonist, liraglutide, has demonstrated the ability to decrease intracellular calcium overload in myocardial injury induced by hydrogen peroxide in vitro [16]. Clinically, liraglutide has been found to improve reperfusion injury in patients with ST-segment elevation myocardial infarction [17]. Both liraglutide [18] and EX-4 [19] protect the heart from cardiac hypertrophy by activating the AMP-activated protein kinase (AMPK) pathway. These findings indicate a potential link between GLP-1 expression and AMPK activation.

Natural products have been identified as potential GLP-1R agonists [20]. Syringaldehyde (SA), a natural phenolic aldehyde with antioxidant properties, has a unique structure consisting of a benzene ring substituted with two methoxy groups and an aldehyde group, making it a promising dual-function GLP-1 receptor modulator [21]. SA has been shown to regulate enzyme activity, protein dynamics, and transcription factors associated with diabetes, inflammation, cancer, and angiogenesis [22,23]. Research indicates that SA improves glucose metabolism and alleviates diabetes through GLP-1 receptor activation [24]. In addition, SA exhibited cardioprotective effects in a myocardial infarction model, indicating its potential in cardiovascular therapy [25]. Similarly, other natural compounds, such as geniposide [26] and catalpol [27], have also demonstrated GLP-1 receptor activation, with geniposide further showing cardioprotective effects by reducing cardiac hypertrophy via the AMPK signaling pathway [19].

This study aims to investigate whether SA can mitigate cardiac hypertrophy under high-glucose conditions, potentially offering new therapeutic strategies for diabetes-related cardiac complications. H9c2 cells, which are immortalized cardiomyocyte-like cells commonly used for in vitro cardiac injury models [28], were employed in this study. Using an established model [24], we examined SA’s effects on glucose-induced cardiac hypertrophy, providing insights into its potential as a GLP-1 receptor agonist in cardiomyocytes [29].

## 2. Results

### 2.1. Syringaldehyde (SA) Activated GLP-1 Receptors in Cardiomyocytes and Alleviated Cardiac Hypertrophy Induced by Hyperglycemia in H9c2 Cells

**Figure 1:**
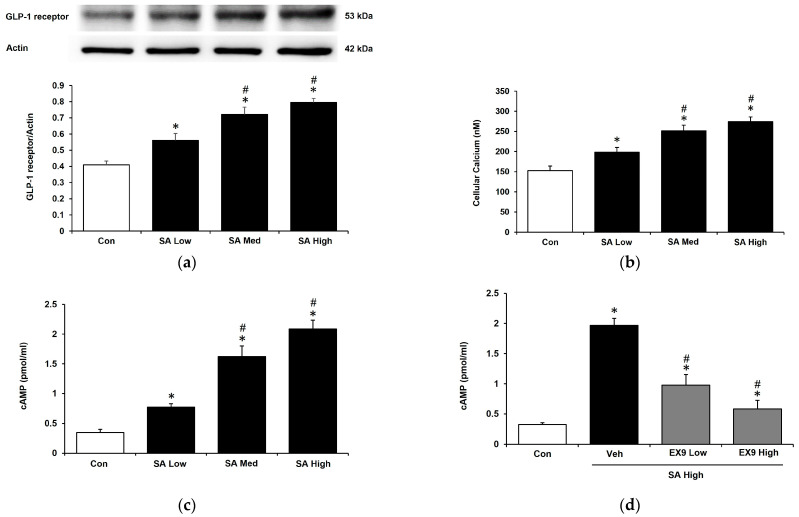
Syringaldehyde (SA) increases the expression of the GLP-1 receptor in H9c2 cells with hypertrophy. (**a**) Changes in GLP-1 receptor protein levels induced via SA administration at concentrations of 0.1 μM (low), 0.5 μM (medium), and 1 μM (high). (**b**) Changes in calcium influx induced via SA administration at different concentrations. (**c**) Levels of cellular cyclic AMP (cAMP) in cells subjected to different concentrations of SA. (**d**) The cellular cAMP level induced by SA at 1 μM was inhibited, following 30 min pretreatment with Ex 9 at concentrations of 0.1 μM or 0.5 μM. The sample size was 6 except for the Western blotting analysis (n = 4). Statistical significance was indicated as follows: * *p* < 0.05 vs. control (Con); ^#^ *p* < 0.05 vs. vehicle (Veh).

SA is recognized to be non-invasive in cardiomyocytes and has been demonstrated to promote the mRNA level of the GLP-1 receptor in H9c2 cells [24]. The concentration of SA treatment was derived from preliminary experiments. SA has increased the protein level of the GLP-1 receptor in a dose-dependent manner in hypertrophic cardiomyocytes as shown by Western blot analysis (Figure 1a). SA treatment also resulted in a concentration increase for both calcium influx and cyclic AMP (cAMP) levels (Figure 1b,c). However, the increase in cAMP levels was inhibited by pretreatment with exendin 9–39 (EX9), a GLP-1 receptor antagonist (Figure 1d). The impact of SA as a GLP-1 receptor agonist was assessed in H9c2 cells.

**Figure 2:**
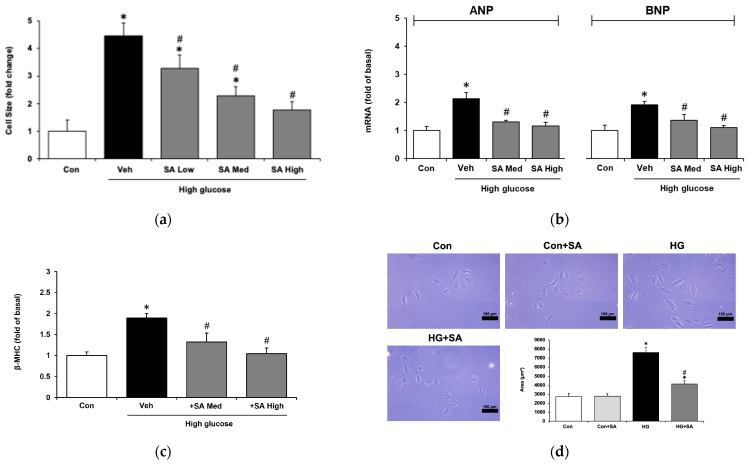
Syringaldehyde (SA) ameliorated the hyperglycemia-induced hypertrophy of H9c2 cells. (**a**) Changes in the size of the hypertrophic cells treated with syringaldehyde at different concentrations of 0.1 μM (low), 0.5 μM (medium), and 1 μM (high). * *p* < 0.05 vs. control (Con). ^#^ *p* < 0.05) vs. vehicle (Veh). Gene expression levels of (**b**) the gene expression levels of ANP (Con: 1 ± 0.2; Veh: 2.1 ± 0.2; SA Med: 1.3 ± 0.1; SA High: 1.2 ± 0.1), BNP (Con: 1 ± 0.2; Veh: 1.9 ± 0.1; SA Med: 1.4 ± 0.2; SA High: 1.1 ± 0.1) and (**c**) β-MHC (Con: 1 ± 0.1; Veh: 1.9 ± 0.1; SA Med: 1.3 ± 0.2; SA High: 1 ± 0.1) in hypertrophic cells administered various concentrations of SA or without treatment. * *p* < 0.05 vs. control (Con). ^#^ *p* < 0.05) vs. vehicle (Veh). (**d**) The size of the H9c2 cells was observed using light microscopy (scale bar: 100 μm). Control cells in a normal culture (Con) were not modified with SA at 1 μM (Con + SA), as indicated in the upper panel. * *p* < 0.05 vs. normal cells (Con). ^#^ *p* < 0.05) vs. high glucose-induced hypertrophic cell (HG). Values for each indicator are expressed as fold changes in gene expressions of ANP, BNP, and β-MHC, relative to the control group. Fold change is calculated as follows: fold change = data of the experimental group/data of the control group.

Hypertrophy was induced in H9c2 cells by incubation in a medium containing 30 mmol/L glucose for 48 h, following the protocol established in our previous study [29]. The morphological changes in cells subjected to various treatments were observed in Figure 2d. High glucose incubation led to a significant increase in cell size and hypertrophic biomarkers, including ANPs, BNPs, and β-MHC. Treatment with SA ameliorated the hypertrophic size in a dose-dependent manner (Figure 2a). Furthermore, the expression levels of hypertrophic biomarkers (ANPs, BNPs, and β-MHC) were significantly reduced, following the administration of SA at medium and high concentrations (Figure 2b,c). These findings suggest that SA exhibits a protective effect against hyperglycemia-induced cardiac hypertrophy in H9c2 cells.

### 2.2. Effects of Syringaldehyde (SA) Were Ablated in H9c2 Cells with GLP-1 Resistance

**Figure 3:**
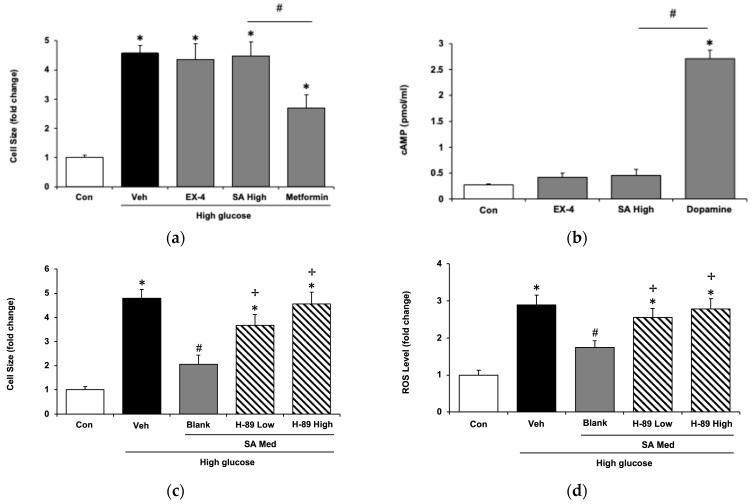
The signaling pathway for syringaldehyde (SA) alleviated cardiac hypertrophy in H9c2 cells. (**a**) Changes in cell size in hypertrophic cardiomyocytes with GLP-1 resistance. Cells were treated with or without SA at 1 μM (high dose), exendin-4 (EX-4) at 0.5 μM, and metformin at 5 μM, respectively. (**b**) SA and EX-4 failed to stimulate cAMP levels in H9c2 cells with GLP-1 resistance, whereas dopamine at 5 μM elevated cAMP levels in these cells. (**c**) A decrease in hypertrophic cell size due to SA at 0.5 μM (a medium dose) was reversed via pretreatment with H-89 at doses of 0.5 μM (low) or 1 μM (high). (**d**) Changes in cellular ROS levels in high glucose-induced hypertrophic cells with or without SA treatment. The inhibition of PKA by H-89 (1 μM) reversed the ROS-lowering effect of SA. Values for each indicator are expressed as fold changes in cell size, gene expressions of ANP, BNP, and β-MHC, relative to the control group. Results are presented as the mean ± SE from independent experiments, n = 6. Statistical significance: * *p* < 0.05 vs. normal control (Con). ^#^ *p* < 0.05 vs. vehicle (Veh). ^†^ *p* < 0.05 vs. hypertrophic cells treated with SA at 0.5 μM (Blank).

Previous studies have suggested that under hyperglycemic conditions, the cardioprotective effects of GLP-1 are significantly impaired, which is referred to as GLP-1 resistance [30]. In the current study, the results demonstrated that the efficacy of EX-4 was markedly diminished in H9c2 cells with GLP-1 resistance (Figure 3a), suggesting a loss of responsiveness to the GLP-1 agonist. Furthermore, SA was unable to ameliorate cardiac hypertrophy in GLP-1-resistant H9c2 cells (Figure 3a). However, as shown in Figure 3a, metformin retained its ability to reduce cell size in hypertrophic cells, even under conditions of GLP-1 resistance.

Additionally, the increase in cAMP due to SA was also ablated in H9c2 cells with GLP-1 resistance (Figure 3b). However, the effect of dopamine on H9c2 cells with GLP-1 resistance was unchanged (Figure 3b). This means that, like exendin-4, the regular GLP-1 receptor is essential to the effect of SA in H9c2 cells.

### 2.3. Protein Kinase A (PKA) Is Involved in the Activation of the GLP-1 Receptor in H9c2 Cells

Protein kinase A (PKA), also known as cAMP-dependent protein kinase, plays a pivotal role in cellular signaling pathways. The cAMP–PKA signaling cascade is critically involved in metabolic regulation [31]. SA may alleviate cardiac hypertrophy in H9c2 cells after GLP-1 receptor activation. To determine whether PKA plays a role in the signaling pathway mediated by SA, we employed H-89, a specific PKA inhibitor, as previously described [32]. As shown in Figure 3c, the effects of SA were dose-dependently reversed by H-89. The dose of H-89 administered was able to inhibit PKA [33]. Interestingly, the same dose of H-89 was able to reverse the reduction in the reactive oxygen species (ROS) by SA in H9c2 cells incubated with high glucose levels (Figure 3d). As in a previous study [34], ROS were increased by high glucose levels in H9c2 cells. SA was found to reduce ROS levels that were increased due to hyperglycemia. ROS were involved in the progression from compensating hypertrophy to heart failure. In the advanced stage of cardiac hypertrophy, ROS induced damage to the myocardium, resulting in myocardial dysfunction or injury [35].

### 2.4. Role of AMPK in Cardiac Effects of Syringaldehyde (SA)

**Figure 4:**
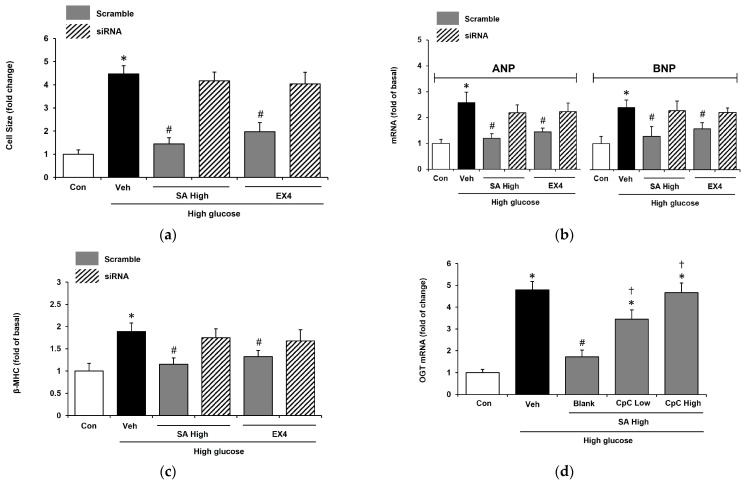
The role of AMPK in syringaldehyde (SA)-alleviated cardiac hypertrophy in H9c2 cells. H9c2 cells were treated with a high glucose medium for 48 h. siRNA was used to ablate the expression of AMPK and H9c2 cells that received with same volume of scramble. (**a**) Changes in cell size due to SA at 1 μM (high) were, like the effect of EX-4, reversed by AMPK ablation. Additionally, the effects of metformin were also removed in AMPK-silenced cells. (**b**) The changes in gene expression levels of ANP (Con: 1 ± 0.2; Veh: 2.6 ± 0.4; SA High + scramble: 1.2 ± 0.2; SA High + siRNA: 2.2 ± 0.3; EX4+ scramble: 1.4 ± 0.1; EX4 + siRNA: 2.2 ± 0.3), and BNP (Con: 1 ± 0.3; Veh: 2.4 ± 0.3; SA High + scramble: 1.3 ± 0.4; SA High + siRNA: 2.3 ± 0.4; EX4+ scramble: 1.6 ± 0.2; EX4 + siRNA: 2.2 ± 0.4). (**c**) The changes in gene expression levels of β-MHC (Con: 1 ± 0.2; Veh: 1.9 ± 0.2; SA High + scramble: 1.2 ± 0.1; SA High + siRNA: 1.7 ± 0.2; EX4+ scramble: 1.3 ± 0.1; EX4 + siRNA: 1.7 ± 0.3). (**d**) The expression of O-linked b-N-acetylglucosamine transferase (OGT) was promoted by high glucose levels and reduced by SA at a 1 μM (high) dose. The effect of SA was reversed by compound C (CpC) at doses of 5 μM (low) and 10 μM (high). The value of each indicator shown in a column is the mean ± standard error of the mean (SEM) per group; n = 6. Values for each indicator are expressed as fold changes in cell size, cAMP levels, and ROS levels relative to the control group. Fold change is calculated as follows: fold change = data of the experimental group/data of the control group. * *p* < 0.05 vs. normal control (Con). ^#^ *p* < 0.05 vs. vehicle (Veh). ^†^ *p* < 0.05 vs. hypertrophic cell administered with SA 1 μM (Blank).

GLP-1R agonist could activate AMPK signaling to prevent glucotoxicity in cardiomyocyte [36]. We applied AMPK siRNA to investigate the role of AMPK in the SA-induced alleviation of cardiac hypertrophy in H9c2 cells. As shown in Figure 4a, as a positive control, exendin-4 failed to modify the size of H9c2 cells in the absence of AMPK. Similarly, SA-induced action in scramble cells was reduced to zero in the absence of AMPK. The same results were observed in metformin-treated H9c2 cells. Moreover, the expression of the hypertrophic gene was changed in the same manner; reductions in the mRNA levels of each gene by SA were ablated in the absence of AMPK, as shown in Figure 4b,c. Finally, the expression of O-linked b-N-acetylglucosamine transferase (OGT) was promoted by high glucose levels, and it was reduced by a 1 μM (high)dose of SA. Interestingly, the effect of SA was reversed by the administration of compound C (Figure 4d) at the dose able to inhibit AMPK [37].

## 3. Discussion

In this study, SA demonstrated the ability to alleviate hyperglycemia-induced cardiac hypertrophy in cardiomyocytes, an effect that appeared to be dependent on the activation of the GLP-1 receptor. Furthermore, SA increased the protein expression level of the GLP-1 receptor in a dose-dependent manner. This was accompanied by a parallel increase in cAMP levels. Notably, the elevation in cAMP levels induced by SA was significantly diminished by the administration of Ex 9-39, a GLP-1 receptor antagonist at a dose sufficient to block receptor activity [38]. These findings indicate that SA activates the GLP-1 receptor in H9c2 cells and exerts protective effects against cardiac hypertrophy.

Myocardial hypertrophy is characterized by an enlargement in cell size and increased expression of embryonic genes, including ANP, BNP, and β-MHC, which can be considered three biomarkers of cardiomyocyte hypertrophy [39,40]. Hypertrophic gene expression in cardiomyocytes is promoted by oxidative stress, insulin resistance, and hyperglycemia [41]. Aortic stenosis, hypertension, and genetic abnormalities, such as hypertrophic cardiomyopathy, may cause maladaptive cardiac hypertrophy, while physiological cardiac hypertrophy is adaptive [42]. Hypertrophic enlargement is characterized by an increase in the cell size of cardiomyocytes [43]. Various sources of ROS have been identified in cardiac cells [42]. LVT hypertrophy (LVH) and heart failure caused by pressure overload have been linked to an increase in oxidative stress and, therefore, an increase in ROS production in the heart. Furthermore, it has been shown that the emergence of cellular remodeling and hypertrophy is linked to increased ROS production. It has been observed that ROS are involved in the progression from compensated hypertrophy to heart failure. It seems that ROS are injurious to the myocardium and cause damage or injury in the latter stages of cardiac hypertrophy [35]. According to the current findings, functional communication between endothelial cells and cardiomyocytes is caused by both direct and indirect processes that result from redox signaling both within and between these cells [44]. However, as including these parameters seemed unnecessary, cultured cardiomyocyte H9c2 cells were used in the present study. A direct reduction in cardiac hypertrophy due to SA was observed in the H9c2 cells. A similar reduction due to SA was also observed in hypertrophic biomarkers, including ANP, BNP, and β-MHC. As a result, SA might have reduced the heart hypertrophy identified in vitro.

The potential mechanism by which SA alleviates cardiac hypertrophy in vitro was examined. A previous study demonstrated that under high-glucose conditions, H9c2 cells exhibit a reduction in GLP-1R expression, resulting in compromised cardioprotective effects of GLP-1 [30]. These cells were subsequently referred to as GLP-1-resistant H9c2 cells [45]. In the present study, the GLP-1-resistant phenotype was confirmed by Western blot analysis, which revealed significantly reduced levels of GLP-1 receptor expression. Although the real mechanism remained unclear, which was likely mediated by Protein Kinase C (PKC) [30] or induced by β-Arrestin [45], this cell model was suitable for examining the role of the GLP-1 receptor. EX-4, an analog of the GLP-1 receptor [15], was able to alleviate cardiac hypertrophy in H9c2 cells after the activation of the GLP-1 receptor [19]. The loss of EX-4 action in this cell model indicated the successful manipulation of the GLP-1 receptor. Interestingly, SA-induced action was also markedly reduced in H9c2 cells with GLP-1 resistance. However, metformin-induced changes were not modified by manipulating the GLP-1 receptor. Additionally, an increase in cAMP due to EX-4 was also reversed in cells with GLP-1 resistance. The same results were observed in SA-treated cells. However, the cAMP-increasing effect of dopamine through another specific receptor [46] was not modified. Therefore, GLP-1 receptor activation via SA is a critical influencing factor that is fully consistent with previous reports [24].

AMPK is a serine–threonine kinase that mainly acts as a metabolic sensor to regulate anabolic and catabolic processes in the heart [47]. In cardiac hypertrophy, AMPK has been suggested to be the key target of agents such as Geniposide [38]. Several upstream signal transduction molecules, including liver kinase B1 (LKB1) and calcium/calmodulin-activated protein kinase kinase 2 (CaMKK2), may activate AMPK through phosphorylation [48]. AMPK has been proposed as an inhibitor of cardiac hypertrophy because it suppresses protein synthesis and ROS generation [49]. The downstream signal pathways of AMPK have been identified as the MAPK family, which includes ERK1/2, p38, and JNK1/2 [50]. The development of pathological cardiac hypertrophy is significantly influenced by these signals [51]. AMPK is a stress-activated kinase [49]. AMPK localized within the intercalated disk of the heart may regulate directional cell migration by modulating the dynamic instability of the microtubule (MT) [52]. Therefore, AMPK has been demonstrated to regulate the cell shape and aspect ratio of cardiomyocytes by modulating the turnover of MTs [53]. This is consistent with the view that the microtubule cytoskeleton is a sensitive target of AMPK [54].

In cardiomyocytes, AMPK is known to reduce ROS production [49] and improve endoplasmic reticulum (ER) stress [55]. Both effects have been widely used to support the role of AMPK in cardiac hypertrophy [38]. AMPK is also known to be linked with autophagy [56]. AMPK may recruit Unc-51-like kinase 1 (ULK1) to activate autophagy [57]. Numerous physiological stressors, including oxidative damage, ER stress, and food constraints, may trigger autophagy, which usually occurs at low basal levels to preserve cellular homeostasis [56]. By removing damaged cells or undesirable macromolecules and organelles, autophagy helps maintain cellular homeostasis, which may negatively impact the structure of the whole heart [58]. Some natural products, such as berberine [59], dihydromyricetin [60], resveratrol [61] and irisin [62], induce autophagy as a mechanism for reducing cardiac hypertrophy. The original objective of autophagy in cardiac tissue was to preserve cardiac structure and function by providing energy, removing ROS, and controlling protein quality to adjust to internal environmental disturbances. However, excessive autophagy harms the heart by increasing cell death [56]. Autophagy modulation has been investigated [63] because higher dosing and long-term treatment with agents may drive excessive autophagy that damages the heart. Therefore, inhibiting excess autophagy using natural products such as Tanshinone IIA [64] has become a popular approach.

**Figure 5:**
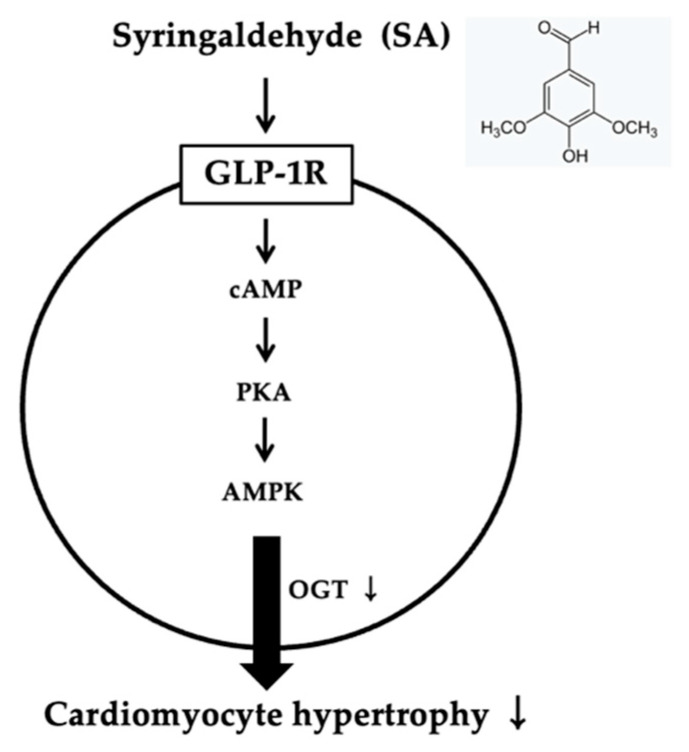
The schematic diagram that presents how syringaldehyde alleviates cardiac hypertrophy.

The association of N-Acetylglucosamine (GlcNAc) with cardiac hypertrophy is widely acknowledged [65]. GlcNAc, an amide derivative of the monosaccharide glucose, is formed through the reaction between glucosamine and acetic acid, and its levels are elevated in cardiac hypertrophy [66]. The modification of serine and threonine residues by GlcNAc, known as O-GlcNAcylation (OGN), is a dynamic process involving the addition and removal of the monosaccharide. This process is mediated by O-linked β-N-acetylglucosamine transferase (OGT) and O-linked β-N-acetylglucosaminase (OGA), respectively [67]. Elevated OGN plays a crucial role in mitigating myocardial remodeling, fibrosis, and hypertrophy induced by short-term exposure to intermittent hypoxia [68]. OGN modulates myocardial hypertrophy through various molecular mechanisms, including the hyperglycemia-induced upregulation of ERK1/2 and cyclin D2 [69]. Additionally, activation of the AMPK pathway has been shown to suppress cardiac hypertrophy in vivo by reducing OGN levels [70]. Therefore, AMPK and OGN are common subjects in research on cardiac hypertrophy. It was recently proposed that SA may improve cardiac hypertrophy in H9c2 cells through AMPK to reduce OGT expression, which is consistent with a recent report that AMPK improved cardiac hypertrophy by reducing OGN [70] (Figure 5).

Compared to other GLP-1R agonists, SA can shorten the α-amylase reaction time and reduce glucose absorption, offering comparable efficacy with potentially fewer side effects [71]. Furthermore, SA has been shown to suppress oxidative stress and inflammatory responses, particularly in myocardial infarction patients, further emphasizing its therapeutic potential and favorable safety profile [72]. Beyond its role in diabetes management, recent studies have also highlighted SA’s anticancer properties, including its ability to inhibit the proliferation of triple-negative breast cancer (TNBC) cells [73]. These findings suggest that exploring SA’s efficacy and safety in animal models of TNBC, as well as its long-term effects on tumor recurrence and metastasis, could be valuable.

In comparison to existing GLP-1 receptor agonists, such as exenatide and liraglutide, SA offers dual functionality by modulating the GLP-1 receptor while simultaneously reducing oxidative stress and inflammation. This makes SA a promising candidate for addressing both glycemic control and the secondary complications of diabetes, such as cardiovascular disease. Unlike synthetic peptide-based agonists that require injection due to their susceptibility to enzymatic breakdown, SA, as a naturally occurring compound, offers the advantages of potentially fewer side effects and broader accessibility when developed as a dietary supplement.

This study is limited to evaluating the effects of SA in hypertrophic H9c2 cells, without incorporating primary cardiomyocytes or in vivo models, which hinders the extrapolation of the results to clinical settings. Additionally, changes in protein levels related to cellular signaling pathways should be examined. Future experiments will systematically collect protein samples for WB analysis to validate findings at the protein level, while studies involving primary cardiomyocytes and in vivo models will also be undertaken. Furthermore, long-term efficacy studies are necessary to assess the sustained therapeutic potential of SA, which will be investigated in subsequent research.

## 4. Materials and Methods

### 4.1. Materials

Syringaldehyde (SA) (purity: 98%), H-89, compound C, exendin-4 (EX-4), exendin 9–39 (EX9), metformin, and other chemicals or reagents were obtained from Sigma-Aldrich (St. Louis, MO, USA).

### 4.2. Culture of Cardiac Cells

The H9c2 cardio myoblast cell line (BCRC No. 60096) was cultured in Dulbecco’s modified Eagle’s medium (DMEM, pH 7.2; Gibco-BRL Life Technologies, Gaithersburg, MD, USA) with 10% fetal bovine serum (FBS, Gibco-BRL Life Technologies, Gaithersburg, MD, USA), in accordance with our previous method [74]. Cells were maintained at 37 °C in a humidified atmosphere with 5% CO_2_. The cells were seeded at a density of 60,000 cells/cm^2^. Cell passage numbers were strictly controlled (passages 3–10) to minimize variability, and a consistent cell source was used throughout the experiments. The medium was replaced on the second day. Experiments were performed in triplicate to ensure reproducibility, and culture conditions were standardized to mitigate variability.

### 4.3. Induction of Cardiac Hypertrophy in H9c2 Cells

A cellular model of cardiac hypertrophy was generated using a high-glucose medium containing 33 mM glucose and 2% FBS. H9c2 cells were incubated with high glucose concentrations for 48 h after achieving 70% confluence, as described previously [29]. D-glucose (Sigma-Aldrich, St. Louis, MO, USA) was dissolved in a normal medium to produce a solution with a concentration of 30 mmol/L glucose. SA was incubated for 1 h before the addition of glucose at a high concentration. The concentrations and treatment durations of SA were determined based on our preliminary experiments [24], which demonstrated their ability to induce significant biological effects while avoiding cytotoxicity. Moreover, our preliminary results showed that H9c2 cells exhibit hypertrophic responses similar to those of primary rat neonatal cardiomyocytes, consistent with previous studies [75]. Thus, the current study utilized H9c2 cells to investigate the hypertrophic response of cardiomyocytes [29].

### 4.4. Induction of GLP-1 Resistance in H9c2 Cells

GLP-1 resistance in H9c2 cells was established following the methodology described in a prior study [30]. H9C2 cells were maintained in either high glucose (HG, 33 mM) or normal glucose (NG, 5 mM) conditions for 21 days, with the culture media replaced every 48 h without subculturing the cells. Hypoxia/re-oxygenation injury in H9C2 cells was induced by subjecting them to hypoxic conditions (95% N_2_, 5% CO_2_) in an anaerobic system (Thermo Forma) at 37 °C for 6 h, followed by 10 h of re-oxygenation under normoxic conditions (95% ambient air, 5% CO_2_). H9c2 cells in a control group were maintained under normal conditions for the same duration. Exendin-4 (EX-4 50 nM) was applied one hour before cell collection to verify the effectiveness of the induction [45]. Depending on the experimental design, syringaldehyde (SA, 0.5 μM or 1 μM), metformin (5 μM), dopamine (5 μM), or H-89 (0.5 μM or 1 μM) were added individually or in combination one hour prior to harvesting the cells.

### 4.5. Detection of Cellular ROS

The intracellular reactive oxygen species (ROS) levels were measured using 2′,7′-dichlorodihydrofluorescein diacetate (DCFH-DA; Sigma-Aldrich), following an established protocol [76]. H9c2 cells were treated with the test compound at a specified concentration for 24 h. After treatment, the cells were washed twice with phosphate-buffered saline (PBS) to remove residual compounds. Subsequently, the cells were incubated with 10 µM DCFH-DA in PBS for 30 min at 37 °C. After incubation, the cells were harvested using trypsin-ethylenediaminetetraacetic acid (EDTA) solution, centrifuged at 200× *g* for 5 min, and resuspended in 0.5 mL of PBS. The fluorescence intensity of the DCF, a marker for ROS levels, was visualized using a fluorescence microscope (Olympus, Tokyo, Japan). Quantification of fluorescence intensity was performed using ImageJ software (version 1.43, Vector Laboratories, Burlingame, CA, USA). The ROS levels were expressed as the ratio of the fluorescence intensity of treated samples to that of control cells, ensuring accurate comparison [77].

### 4.6. Cardiomyocyte Transfections

To investigate the interaction between SA and AMPK signaling pathways, H9c2 cells were first induced to develop hypertrophy. Subsequently, siRNA transfections were conducted as previously described [78]. The transfection reagent and siRNAs targeting AMPK (GCATATGCTGCAGGTAGAT) were obtained from Dharmacon (Horizon Discovery, Lafayette, CO, USA). Cells were transfected with either AMPKα1-specific siRNA (100 nM) or an equivalent amount of scrambled siRNA using Lipofectamine 2000 (0.15%, *v*/*v*, Thermo Fisher Scientific, Waltham, MA, USA) for 12 h. After that, lipofectamine 2000 was removed by changing into a fresh medium containing 10% FBS. The cells were then administered with drugs and further analysis following transfection.

### 4.7. Quantitative Reverse-Transcription Polymerase Chain Reaction (qRT-PCR) of O-Linked N-Acetylglucosamine Transferase (OGT)

As with the biomarkers of hypertrophy, ANP, BNP, and β-MHC [29], the expression of OGT was determined via qRT-PCR as described in our previous report [24]. Total RNA was extracted by using a TRIzol reagent (Invitrogen, Carlsbad, CA, USA). qRT-PCR primers were acquired from Roche (Roche Diagnostics GmbH, Mannheim, Germany). Approximately, 200 ng of total RNA was reverse-transcribed into cDNA. As in the previous study [78], the primers used in the experiment were as follows:
*ANP*F: 5′-CACAGATCTGATGGATTTCAAGA-3′;
R: 5′-CCTCATCTTCTACCGGCATC-3′;*BNP*F: 5′-GTCAGTCGCTTGGGCTGT-3′;
R: 5′-CCAGAGCTGGGGAAAGAAG-3′;*β-MHC*F: 5′-CATCCCCAATGAGACGAAGT-3′;
R: 5′-GGGAAGCCCTTCCTACAGAT-3′*Ogt*F: 5′-G GCTATGTGAGTT CTGACTTCGG-3′ 
R: 5′-GATTGGCTTCCGCCATCACCTT-3′,*Gapdh*F: 5′-CATCACTGCCACCCAGAAGACTG-3′ 
R: 5′-ATGCCAGTGAGCTTCCCGTTCAG-3′ 

The concentration of each qRT-PCR product was determined by comparison with a corresponding standard curve to ensure quantitative accuracy [24].

### 4.8. Western Blot Analysis

H9C2 cells subjected to various treatments were harvested and lysed for Western blot analysis [24]. Primary antibodies used were anti-GLP-1R (1:200, Abcam, Cambridge, UK) and anti-β-Actin (1:5000, Abcam, Cambridge, UK) as a loading control. Membranes were incubated with the primary antibodies overnight at 4 °C. After washing with TBST, the membranes were incubated with horseradish peroxidase-conjugated goat anti-rabbit IgG secondary antibodies (1:5000, Santa Cruz, CA, USA) for 1 h at room temperature. Protein bands were visualized using an enhanced chemiluminescence (ECL) detection system (Amersham, UK). Densitometric analysis of the protein bands was conducted using ImageJ software (version 1.43; NIH, Bethesda, MD, USA) to quantify relative protein expression levels.

### 4.9. Detection of Intracellular Calcium Level

The intracellular calcium concentration [Ca^2+^]i changes were measured using the fluorescent probe fura-2, as previously detailed [79]. Cells were harvested and suspended in PBS, after which 5 μM Fura-2 was added for staining. Following incubation, the fluorescence intensity was measured using a fluorescence spectrofluorometer (Hitachi F-2000, Tokyo, Japan). Background fluorescence from unloaded cells was subtracted from all measurements to ensure accuracy. The intracellular calcium concentration was then calculated according to the previous study [79].

### 4.10. Statistical Analysis

For each experimental group, results are presented as the mean ± standard error of the mean (SEM) based on the designated sample size. Data were analyzed using SPSS statistical software version 21 (SPSS Inc., Chicago, IL, USA). An unpaired *t*-test was used to assess the differences between the two groups, while a one-way analysis of variance (ANOVA) followed by Tukey’s post hoc test was applied to compare differences among more than two groups. Statistical significance was established at *p* < 0.05.

## 5. Conclusions

Syringaldehyde demonstrates the potential to attenuate hyperglycemia-induced cardiac hypertrophy in H9c2 cells, primarily by activating the GLP-1 receptor. This mechanism involves the AMPK signaling pathway and the suppression of OGT expression. This study represents a preliminary investigation into the therapeutic potential of SA. Overall, this study highlights the potential of SA to advance herbal medicine and serve as a novel therapeutic strategy for managing cardiac hypertrophy in diabetes.

## Data Availability

The data presented in this study are available from the corresponding author upon reasonable request.
